# Estimating the Photorespiratory CO_2_ Compensation Point and CO_2_ Release in the Light Using the Laisk Method Combined With Photosynthetic Theory

**DOI:** 10.1111/pce.70195

**Published:** 2025-09-23

**Authors:** Darwin L. Moreno‐Echeverry, Miko U. F. Kirschbaum, Margaret M. Barbour, Lìyǐn L. Liáng

**Affiliations:** ^1^ Bioeconomy Science Institute Manaaki Whenua Landcare Research Palmerston North New Zealand; ^2^ Te Aka Mātuatua—School of Science University of Waikato Hamilton New Zealand

**Keywords:** CO_2_ compensation point, day‐respiration, FvCB model, gamma‐star, photosynthesis, R_day_, R_L_, temperature‐response

## Abstract

The photorespiratory CO_2_ compensation point (Γ*) and the rate of CO_2_ release in the light (D_L_) are critical parameters for understanding the carbon dynamics of C_3_ plants. These two parameters can be derived from the widely‐used Laisk method as the intercept of linear regression lines fitted to net assimilation rates (A_net_) vs. chloroplast CO_2_ partial pressures (C_c_) obtained at different low‐irradiance levels. However, photosynthetic theory indicates curvature in the A_net_‐C_c_ relationship which conflicts with the use of linear relationships for analysis. We, therefore, systematically evaluated the limitations of the use of linear relationships across temperatures from 5°C to 40°C and quantified the sensitivity of errors in Γ* and D_L_ estimates to the selected C_c_ range. We found that wide CO_2_ ranges, especially when they exclude the expected Γ*, can introduce substantial biases in parameter estimation with linear regressions, particularly at lower temperatures. This can lead to marked underestimates of Γ*, and biologically unrealistic D_L_. We propose refining the Laisk method by using a photosynthesis model to analyse data. The model better represents the nonlinear A_net_‐C_c_ relationship and yields consistent Γ* and D_L_ estimates, regardless of the CO_2_ range used.

## Summary statement

- The photosynthetic parameters Γ* and CO_2_ release in the light can be estimated using linear relationships with the Laisk Method.
- We analysed the errors inherent in that approach and described an alternative by combining the Laisk Method with photosynthetic modelling for better parameter estimates.

## 1 Introduction

Plants assimilate carbon from the atmosphere through photosynthesis. This physiological process can be described using mathematical models, among which the model developed by Farquhar, von Caemmerer, and Berry (FvCB model) in 1980 is the most widely used for understanding and quantifying carbon fixation in terrestrial C_3_ plants (Farquhar et al. 1980; Walker and Ort 2015; Herrmann et al. 2020; Machino et al. 2021). The FvCB model includes key biochemical parameters that are essential for predicting plant responses to environmental changes (Farquhar et al. 1980; Ubierna et al. 2019) and plays a crucial role in assessing carbon balances from individual leaves to global ecosystems (Huntingford et al. 2017; Kitao et al. 2021; Sun et al. 2014; Walker et al. 2017).

Among the parameters of the FvCB model, two important ones are the photorespiratory CO_2_ compensation point (Γ*) and the rate of decarboxylation in the light (D_L_), also known as the rate of light respiration (R_L_) or day respiration (R_day_) (Berghuijs et al. 2019; Gong et al. 2018; Schmiege et al. 2023; Walker and Cousins 2013). We here refer D_L_ as the total CO_2_ release under light to capture the CO_2_ contributions of respiratory processes and decarboxylation processes following the terminology used in Tcherkez et al. (2024). D_L_ only excludes CO_2_ released by photorespiratory decarboxylation.

Accurate estimation of D_L_ is critical for understanding carbon dynamics from individual leaves to ecosystem scale, and for assessing global carbon budgets (Heskel 2018; Needham et al. 2025; Ranathunga et al. 2025; Smith and Dukes 2013). However, quantifying D_L_ remains an experimental challenge (Kroner and Way 2016; Way et al. 2019), because D_L_ cannot be measured directly using gas exchange techniques. It must be estimated, with the method developed by Laisk (1977), as described by Brooks and Farquhar (1985), being one of the most widely applied approaches (Berghuijs et al. 2019; Gong et al. 2018). In addition, other approaches such as the Kok and Yin methods are widely discussed alternatives (Yin et al. 2011; Farquhar and Busch 2017; Tcherkez et al. 2017). However, these methods are used exclusively to estimate D_L_, and cannot be applied to determine Γ*. Several studies have also reported that D_L_ is consistently underestimated when using the Kok method, especially under photorespiratory conditions (Yin and Amthor 2024) when the inevitable changes in intercellular CO_2_ concentrations under changing light levels are ignored (Kirschbaum and Farquhar 1987).

The Laisk method involves estimating Γ* and D_L_ by plotting measurements of photosynthesis (A_net_) against low CO_2_ partial pressure, measured under different levels of photosynthetic photon flux density (PPFD) (Berghuijs et al. 2019; Laisk 1977; Walker and Cousins 2013). Linear regressions are then applied to subsets of these points, and the intersection of the resulting lines is used to infer Γ* and D_L_. However, according to the theory outlined in the equations of the FvCB model, the response of photosynthesis to CO_2_ is inherently curvilinear (Farquhar et al. 1980; Walker and Ort 2015), meaning that the linear approximation introduces systematic errors in the estimation of both parameters.

Some strategies have therefore been proposed to minimise the biases associated with this linear approximation. One such strategy is the slope–intercept regression method, a refinement of the common intercept approach that does not directly address the physiological nonlinearity, but instead reduces bias caused by similar slopes between neighbouring lines. This method assigns greater weight to intersections where the slopes differ more markedly, thereby improving the robustness of the linear fit (Walker and Ort 2015). Alternatively, some studies have applied polynomial regressions to capture the curvature more explicitly (Tholen et al. 2012; Walker and Ort 2015). However, polynomials have no theoretical or physiological basis for describing photosynthesis, and their use can lead to unrealistic values for Γ* and D_L_ if any extrapolation is needed, or even at the extreme points of the available observations.

More recently, Yin and Amthor (2024) proposed an alternative to the linear Laisk approach by applying a non‐rectangular hyperbolic model to simultaneously estimate D_L_ and mesophyll conductance (g_m_). Their method incorporates chlorophyll fluorescence to account for the variation of electron transport with light intensity, and explicitly corrects for the CO_2_ reassimilation that occurs during measurements at low CO_2_ (Yin and Amthor 2024). Use of the non‐rectangular hyperbolic model leads to D_L_ estimates that are approximately 25% higher than those obtained with the traditional Laisk method, highlighting the importance of incorporating nonlinear analysis into the method.

**Figure 1:**
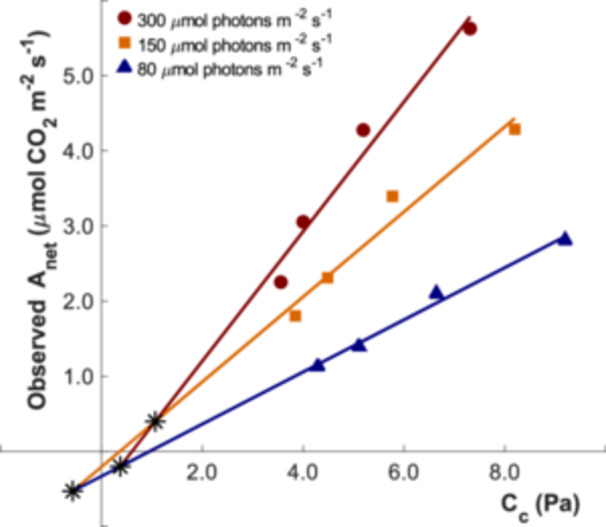
Observed net photosynthetic rate (A_net_) as a function of chloroplastic CO_2_ partial pressure (C_c_), measured at 6°C under three light intensities: 300, 150, and 80 µmol photons m^−2^ s^−1^ (shown in the dark red, orange and blue, respectively). The CO_2_ partial pressure ranged from 3.6 to 9.2 Pa. Experimental data points are shown individually, with linear regressions fitted for each light level (solid lines). The intersections of the regression lines are marked with black asterisks.

Estimating Γ* and D_L_ under low‐temperature conditions using the Laisk method can be problematic and may lead to erroneous results, particularly if equipment constraints prevent the use of CO_2_ partial pressures close to the expected Γ* (Γ*_exp_) (Atkin et al. 2000; Tcherkez and Atkin 2021). In that case, the linear regression approach may yield erroneous values of D_L_, as shown in the intersection of the regression lines for 300 and 150 µmol photons m^−2^ s^−1^ (Figure 1). These findings highlight the limitations of the Laisk method in accurately estimating Γ* and D_L_, especially at low temperatures (Atkin et al. 2000; Way et al. 2019).

Way et al. (2019) attributed the occurrence of erroneous negative D_L_ values to factors such as low‐temperature effects on chloroplast CO_2_ partial pressure, cuvette leaks, and anaplerotic CO_2_ fixation. However, the primary cause of these inaccuracies could also arise from the nonlinear nature of A_net_‐C_c_ curves. When this nonlinearity varies with the selection of a CO_2_ partial pressure range that does not include a Γ*_exp_ value, it can lead to physiologically impossible values of D_L_, particularly at low temperatures. This highlights the need for an approach capable of estimating accurate and physiologically meaningful D_L_, to produce high‐quality data that can support robust interpretations of carbon gains and losses in plants, and improve the representation of plant carbon balance under light conditions in ecosystem and global models.

In this study, we test the hypothesis that the range of CO_2_ partial pressure used in the Laisk method significantly affects the estimation of Γ* and D_L_ due to the underlying nonlinear nature of the A_net_‐C_c_ relationship. Using both simulated and experimental data, we quantify the biases introduced by applying linear regressions, comparing the results to theoretical expectations based on photosynthetic theory. We further examine how the choice of CO_2_ range influences the accuracy of these estimates across temperatures. Based on these findings, we propose a refined methodology that improves the accuracy of photosynthetic carbon dynamic predictions across varying environmental conditions by applying the FvCB model to experimental datasets.

### 1.1 Theory

The FvCB model of C_3_ photosynthesis consists of three components that together determine the CO_2_ exchange at the leaf level (Farquhar et al. 1980): the carboxylation by Rubisco (gross photosynthesis), the oxygenation by Rubisco (photorespiration), and D_L_ (CO_2_ released in the light from processes other than photorespiration) (Ubierna et al. 2019; Tcherkez et al. 2024). While it was traditionally believed that CO_2_ release under light conditions was exclusively due to respiratory processes, it is now understood that anabolic pathways also contribute significantly (Abadie et al. 2024; Tcherkez et al. 2024; Tcherkez and Atkin 2021). These include the decarboxylation of pyruvate during fatty acid biosynthesis, the conversion of glucose‐6‐phosphate to ribose‐5‐phosphate via the pentose phosphate pathway for sucrose production, and the decarboxylation of isocitrate to 2‐oxoglutarate (2‐OG) during amino acid biosynthesis (Abadie et al. 2024; Tcherkez et al. 2024). Gas exchange methods estimate the total CO_2_ release under light conditions and therefore cannot differentiate between the contributions of respiratory processes and other decarboxylation processes, making the use of terms like R_day_ or R_L_ conceptually misleading. By incorporating these metabolic processes into the definition of D_L_, we aim to more accurately capture the full scope of CO_2_ released in the light.

Net photosynthesis can be calculated in terms of the rate of Rubisco carboxylation, as:

(1) $$A_{\mathrm{net}}=V_{c}\;\left(1-\Gamma^{*}/C_{c}\right)-D_{L}$$

where V_c_ is the rate of carboxylation, Γ* is the photorespiratory CO_2_ compensation point and C_c_ is the chloroplast CO_2_ partial pressure. V_c_ can be determined as the minimum rate of three possible limiting factors: the Rubisco‐limited carboxylation rate (W_c_), the carboxylation rate limited by RuBP (Ribulose 1,5‐bisphosphate) regeneration or electron transport rate (W_j_), and the carboxylation rate constrained by phosphate availability (W_p_). Therefore, at a given chloroplastic CO_2_ partial pressure, A_net_ is modelled as the minimum of these three limiting rates (W_c_, W_j_, W_p_) as shown in Equation 2 (Kirschbaum and Farquhar 1984; Ubierna et al. 2019).

(2) $$V_{c}=\min\{W_{c},W_{j},W_{p}\}$$

Since W_p_ was not limiting under our experimental conditions, this term can be omitted. W_c_ is calculated as:

(3) $$W_{c}=\frac{V_{\mathrm{cmax}}^{\prime }\times C_{c}}{C_{c}+K_{m}}$$

where ${V^{\prime }}_{\mathrm{cmax}}$ is the apparent maximum carboxylation rate and K_m_ is the effective Michaelis–Menten constant for CO_2_ that also takes into account competitive inhibition by oxygen. In the calculation of K_m_, the partial pressure of oxygen (O_2_), and the Michaelis–Menten constants for CO_2_ (K_c_) and for O_2_ (K_o_) are incorporated as shown in Equation 4.

(4) $$K_{m}=K_{c}\left(1+\frac{O_{2}}{K_{o}}\right)$$

${V^{\prime }}_{\mathrm{cmax}}$ is calculated as:

(5) $${V^{\prime }}_{\mathrm{cmax}}=\frac{V_{\mathrm{cmax}}\times C_{c}}{C_{c}+K_{a}}$$

where V_*c*max_ is the maximum carboxylation rate, and K_a_ is the Michaelis–Menten constant for activation of Rubisco by CO_2_ at a given Mg^2+^ concentration and pH (Kirschbaum and Farquhar 1987).

W_j_ is calculated as:

(6) $$W_{j}=\frac{J\;*\;C_{c}}{4\left(C_{c}+2\Gamma^{*}\right)}$$

where J is the potential electron transport rate. We used the factor of 2 here instead of 7/3 by assuming that NADPH reduction rather than ATP production, is the limiting step in the regeneration of RuBP (Kirschbaum and Farquhar 1987). The factor 4 converts electron transport into RuBP‐regeneration capacity because one molecule of RuBP requires two molecules of NADPH, each of which requires two electrons.

J is calculated as:

(7) $$J=\frac{\left(\alpha \;*\;I+J_{\max}\right)-\sqrt{{\left(\alpha \;*\;I+J_{\max}\right)}^{2}-4\theta \;\left(\alpha \;*\;I\;*\;J_{\max}\right)}}{2\theta }$$

where J_max_ is the maximum electron transport rate, *α* is the quantum yield of electron transport, I is the photon flux density of photosynthetically active radiation and θ describes the curvature of the light response of photosynthesis (Buckley and Farquhar 2004).

However, the transition between these limitations on V_c_ is not as abrupt as implied by Equation 2. The intrinsic nature of enzymatic kinetics and the variability in leaf tissue properties result in a region on the A_net_‐C_c_ curve where different factors can co‐limit the overall rates (Kirschbaum and Farquhar 1984) as multiple processes simultaneously restrict the current rate of photosynthesis. Consequently, Equation 2 was rearranged by introducing an empirical factor (*e*), which transforms the function into a smooth hyperbola (Equation 8).

(8) $$V_{c}^{2}-V_{c}(W_{c}+W_{j}+e)+W_{c}W_{j}=0$$

Under our light regime the A_net_‐C_c_ curves are predominantly RuBP‐regeneration limited, so *e* has minimal leverage on parameter estimation; varying *e* from 0.00 to 1.00 altered Γ* and D_L_ by less than 1% (data not shown). By rearranging Equation (8), the transition between the limitation of the carboxylation rate due to both Rubisco and RuBP regeneration can be obtained, as shown in Equation (9).

(9) $$V_{c}=\frac{\left(W_{c}+W_{j}+e\right)-\sqrt{{\left(W_{c}+W_{j}+e\right)}^{2}-4\left(W_{c}W_{j}\right)}}{2}$$

The resulting V_c_ from Equation 9 is then substituted into Equation 1. The estimated assimilation rate is thus calculated based on the FvCB model, incorporating modifications proposed by Kirschbaum and Farquhar (1984), and further refinements by Ubierna et al. (2019).

## 2 Materials and Methods

### 2.1 Growth Conditions and Experimental Data Collection

Sunflower (*Helianthus annuus* “Russian Giant”) plants were grown from seed in 1.5 L plastic pots and cultivated in a plant growth unit. Throughout the experiment, plants were well‐watered and fertilised regularly. Day/night temperature and relative humidity were 24/19°C and 40/65%, respectively. Photoperiod was 14 h with a light intensity of 400 µmol photons m^−2^ s^−1^ at the leaf level. Fully expanded leaves from the upper canopy were selected for CO_2_ assimilation measurements. These measurements were performed using a GFS‐3000 portable gas‐exchange and fluorescence system (Heinz Walz GmbH, Effeltrich, Germany), equipped with a 4 cm² leaf chamber and a red‐blue LED light source (10% blue, 90% red).

Before determining assimilation rates at each light intensity and CO_2_ partial pressure, the leaves were given a 30‐min metabolic adjustment period at 6°C, under a CO_2_ partial pressure of 40 Pa and 300 µmol photons m^−2^ s^−1^. This period ensured that photosynthesis had reached a steady state. Similarly, following each change in light intensity, the CO_2_ partial pressure was set to 40 Pa to ensure full Rubisco activation.

Measurements were taken at a range of CO_2_ partial pressures (1.2 to 9.2 Pa) under three photon flux densities: 300, 150, and 80 µmol photons m^−2^ s^−1^. For subsequent analysis, the data set was divided into four subsets, based on the inclusion or exclusion of Γ*_exp_, estimated at 1.54 Pa at 6°C (Brooks and Farquhar 1985). For subsets where Γ*_exp_ was included, the CO_2_ ranges were 1.2–3.6 Pa (narrow) and 1.2–6.6 Pa (wide). For those excluding Γ*_exp_, the narrow range was 3.6–6.6 Pa and the wide range was 3.6–9.2 Pa.

### 2.2 Definitions of Γ* Used in This Study

In this study, we deal with the empirical derivation of Γ* from gas‐exchange measurements. We outline theoretical problems that arise from the derivation of Γ* from gas exchange data and how they relate to the range of CO_2_ partial pressures available for analysis. For that, we needed to distinguish between Γ* with three possible meanings. To avoid confusion in the following text, they are briefly outlined here:

- Γ* (Estimated Γ*): The actual value estimated directly from available experimental data. That is the value one aims to refine through additional measurements.
- Γ*_ref_ (Reference Γ*): The value used in our illustrative modelling to highlight problems that arise from using different approaches to derived Γ* at different temperatures and over different ranges of CO_2_ partial pressures.
- Γ*_exp_ (Expected Γ*): The value of Γ* broadly expected at specific temperatures. It would be the value that can typically be used in experimental work to define an appropriate CO_2_ range for measurements so that Γ* falls within, or, at least, close to the range of measurements.

Γ*_ref_ and Γ*_exp_ are values obtained either from established empirical equations in the literature (e.g., Brooks and Farquhar 1985) or calculated from the partial pressure of oxygen and the specificity of Rubisco.

### 2.3 Estimation of Γ* and D_L_ Using the Laisk Method

Γ* and D_L_ were estimated using the traditional Laisk method, which involves determining the intersection points between pairs of linear regressions fitted to data obtained at different light intensities. When more than two light levels are available, multiple pairwise intersections are calculated, and the final estimates of Γ* and D_L_ are obtained by averaging the *x*‐ and *y*‐coordinates of all intersection points.

The *x*‐coordinate of each pairwise intersection point, representing an individual Γ* estimate, was calculated as:

(10) $$\Gamma_{\mathrm{ij}}^{*}=\frac{b_{i}-b_{j}}{m_{j}-m_{i}}$$

where *m*_*i*_ and *m*_*j*_ are the slopes, and *b*_*i*_ and *b*_*j*_ are the *y*‐intercepts, of the regression lines corresponding to light levels *i* and *j*. Similarly, the corresponding *y*‐coordinate for each intersection, representing an individual D_L_ estimate, was calculated as:

(11) $$D_{L\mathrm{ij}}={m_{i}\Gamma }_{\mathrm{ij}}^{*}+b_{i}$$

The final values of Γ* and D_L_ were then determined by averaging all pairwise estimates:

(12) $$\Gamma^{*}=\frac{1}{n}\sum_{\left(i,j\right)}\Gamma_{\mathrm{ij}}^{*}$$

(13) $$D_{L}=\frac{1}{n}\sum_{\left(i,j\right)}D_{L\mathrm{ij}}$$

Where *n* is the total number of pairwise intersections.

### 2.4 Common Considerations in Laisk Analyses: Environmental Dependence of g_m_ and D_L_

An important methodological consideration in applying the FvCB model is that the estimation of Γ* and D_L_ must be based on chloroplastic CO_2_ partial pressure (C_c_), rather than intercellular CO_2_ partial pressure (C_i_). The Laisk method was originally developed under the implicit assumption of infinite g_m_, implying C_i_ = C_c_. However, it is now well established that g_m_ is smaller than originally assumed and typically small enough to require an important additional diffusion gradient between the intercellular air spaces and the chloroplastic sites of photosynthesis.

The mesophyll conductance is sensitive to multiple environmental factors (Bernacchi et al. 2002; Flexas et al. 2007; Li et al. 2020; Shrestha et al. 2019). In this context, g_m_ functions as a dynamic variable, co‐varying with other photosynthetic traits in response to light, temperature, and CO_2_ regimes. Supporting Information S2 Figure S1 presents a set of experimental results demonstrating the environmental sensitivity of g_m_ in sunflower (*H. annuus*). Mesophyll conductance was quantified using a combined gas exchange and carbon isotope discrimination technique, and its response was characterised across gradients of light intensity, leaf temperature, and intercellular CO_2_. These data showed that g_m_ is not constant but may vary meaningfully with external conditions.

The question then arises whether variability and uncertainty in g_m_ can lead to significant errors in Γ* and D_L_ estimates. To explore this, and to test the robustness of Γ* and D_L_ estimates with respect to g_m_, we conducted a comprehensive sensitivity analysis (Supporting Information S2: Figure S2, using the Excel tool developed in this study (Supporting Information S1: File Excel Template). We varied g_m_ from 0.5 to 10.0 µmol m^−^² s^−^¹ Pa^−^¹ at 25°C. By spanning this extensive range, the analysis captured the potential variability in g_m_ across different plant functional types and environmental contexts.

The results of this sensitivity analysis showed that both Γ* and D_L_ estimates remained remarkably stable across this range, particularly within the 2–10 µmol m⁻² s⁻¹ Pa⁻¹ interval, where Γ* was 3.67 Pa and D_L_ was 1.27 µmol CO_2_ m⁻² s⁻¹. Even at the lowest conductance (0.5 µmol m⁻² s⁻¹ Pa⁻¹), where variation was most pronounced, Γ* increased by only 0.8% (from 3.67 to 3.70 Pa) and D_L_ by only 3.1% (from 1.27 to 1.31 µmol CO_2_ m⁻² s⁻¹). This outcome highlights the robustness of the method even when g_m_ is variable or uncertain, because measurements made near Γ* involve very low net CO_2_ assimilation rates. Mesophyll diffusion gradients between intercellular air spaces and chloroplasts are therefore minimal (Walker and Ort 2015; Bush et al. 2024). In addition, since the Laisk method derives Γ* and D_L_ from the intercept of multiple A_net_‐C_c_ regressions, systematic variations in CO_2_ drawdown across treatments are largely compensated in the intercept calculation. Even at slightly higher CO_2_ partial pressures and larger A_net_ rates, the chloroplastic CO_2_ drawdown required to overcome mesophyll diffusion limitations is proportional to CO_2_ uptake, so its effect cancels when the intercepts of multiple lines are used to derive Γ* and D_L_, because the intercept reflects a condition where A_net_ approaches zero.

A second issue in the classical Laisk method it is commonly assumed that D_L_ remains constant across changes in irradiance and CO_2_ during the experiment. From a methodological standpoint, reports of CO_2_ dependent changes in leaf respiration may partly reflect gas exchange artefacts (cuvette leakage, calibration drift or lack of steady state), rather than metabolic regulation, which cautions against over‐interpreting CO_2_ effects on D_L_ within our conditions (Amthor et al. 2001; Yin and Amthor 2024). However, consideration of possible artefactual explanations does not preclude the possibility that there may also be genuine metabolic variability. Recent metabolomic and isotopic evidence has shown that CO_2_ release in the light draws on multiple carbon sources, including stored substrates that could lead to short‐term dependence on recent CO_2_ and irradiance (Abadie et al. 2024). Over the irradiance range commonly used for gas‐exchange step measurements, the fraction of dark respiration that persists in the light is practically unaffected by irradiance (Kirschbaum and Farquhar 1987; Peisker and Apel 2001). Accordingly, we treat D_L_ as invariant in the present analyses, while noting that any potential dependence on CO_2_ and low irradiance should be tested explicitly in future work.

### 2.5 Simulations for Quantifying the Bias of Laisk Method

To quantify the bias of the estimated Γ* and D_L_ values using the Laisk method across different temperatures, we simulated net assimilation rates (A_net_) using the FvCB model, as described in Section 1.1, across a temperature range from 5°C to 40°C and under three light intensities (300, 150, and 80 µmol photons m^−2^ s^−1^). Parameters used in the simulation are detailed in the Supporting Information S2: Table S1. V_cmax_ and J_max_ were derived from experimental measurements at 25°C and their temperature dependence was modelled using the MMRT function (Arcus et al. 2016; Liang et al. 2018) (see equations in Supporting Information S2: Notes S1). *α* and θ were kept constant across all temperatures.

For each light level, we simulated A_net_ under three C_c_ levels that were varied under different temperatures and were defined relative to Γ*_ref_ (the reference value of Γ*) calculated as: Γ*_ref_ + 2.1 Pa, Γ*_ref_ + 4.6 Pa, and Γ*_ref_ + 7.2 Pa. Γ*_ref_ was calculated following the equation described by Brooks and Farquhar (1985). By varying the C_c_ level under different temperatures that corresponded to Γ*_ref_, we avoided any potential bias from mismatches between Γ* and the CO_2_ range to better quantify the effectiveness of the Laisk method on estimating Γ* and D_L_. At every temperature under the selected light levels, that is, 300, 150, and 80 µmol photons m^−2^ s^−1^, we constructed three A_net_‐C_c_ curves that intersected in a unique point, which was the prescribed Γ* in our simulation (Γ*_ref_). Based on the simulated A_net_ at each temperature, we applied the Laisk method to estimate Γ* and D_L_ using linear regression outlined above. The differences between the derived Γ* and Γ*_ref_ under temperatures from 5°C to 40°C denoted the bias in estimating Γ* using the Laisk method as Є(Γ*). A bias estimate for DL, Є(D_L_), was derived similarly.

To better understand the effect of different ranges of C_c_ on the estimation of Γ* and D_L_, we conducted a sensitivity analysis by extending our simulations using two distinct C_c_ ranges: inclusion of Γ*_ref_ and exclusion of Γ*_ref_ within the selected C_c_ ranges. Both scenarios, in which Γ*_ref_ was either included or excluded from the selected C_c_ range, were evaluated across the temperatures ranging from 5°C to 40°C. When Γ*_ref_ was included, each C_c_ range began 0.5 Pa below Γ*_ref_ with the initial range extending to 0.5 Pa above it. The upper limit of each subsequent range was extended by 0.5 Pa, up to a maximum of 10 Pa. In contrast, when Γ*_ref_ was excluded, the C_c_ range began at 3.0 Pa above Γ*_ref_. The first range extended 1.0 Pa above that starting point, and, similarly, the upper limits of subsequent ranges were increased by 0.5 Pa until a maximum of 10.5 Pa was reached.

### 2.6 Simulations of Modelled Intercepts for Linear Regressions and FvCB Model

Except for bias introduced by the Laisk method, measurement errors from infrared gas analysers (IRGAs) can also contribute to errors in estimating Γ* and D_L_. Based on experimental data collected under varying conditions of temperature, light, and CO_2_ partial pressure, we determined a standard deviation of 0.07 µmol CO_2_ m⁻^2^ s⁻^1^ in the net assimilation rates of our gas‐exchange systems (data no shown).

To incorporate this uncertainty, we implemented a Monte Carlo approach, generating 10,000 A_net_ simulated values per treatment. Each treatment corresponded to a specific combination of CO_2_ range (narrow or wide) and inclusion or exclusion of Γ*_ref_ in the analysed C_c_ interval. Simulated A_net_ values (A_sim_) were produced using the Box–Muller transformation (Scott 2011), which converts two uniformly distributed random numbers (u_1_, u_2_) into a normally distributed variable (z) with a mean of 0 and a standard deviation of 1, according to the following equation:

(14) $$z=\sqrt{-2\mathrm{Ln}\left(u_{2}\right)}\;*\;\cos\left(2\pi \;*\;u_{1}\right)$$

For each data set, the A_sim_ was computed using the equation:

(15) $$A_{sim}=A_{\mathrm{obs}}+z\left(i\right)\;*\;\text{error}$$

where z(i) represents the i‐th value generated through the Box‐Muller method, A_obs_ is the observed value of A_net_, and *error* denotes the standard deviation of the measurements, that is, 0.07 µmol CO_2_ m⁻^2^ s⁻^1^. This approach allowed us to incorporate measurement uncertainty and evaluate its effect on the estimation of Γ* and D_L_ using both linear regression and the FvCB model.

### 2.7 Application of the Photosynthetic Model to Experimental Data

We applied the photosynthetic model to the experimental data collected during the first phase of the study. The fitting process involved optimising the parameters V_cmax_, J_max_, *α*, and θ, as well as estimating Γ* and D_L_, using the parameter values listed in Supporting Information S2: Table S1. Parameter optimisation was performed in MATLAB using the *fminsearch* function to minimise the negative log‐likelihood between observed and modelled net assimilation rates. Through iterative adjustment of the model parameters, this procedure yielded the best‐fitting values for each treatment, resulting in a unique intersection point among the three A_net_‐C_c_ curves (Supporting Information S2: Notes S2).

To facilitate broader application and reproducibility of the model‐fitting approach, we also developed an interactive Excel‐based tool (Supporting Information S1: File Excel Template). This tool implements the same optimisation framework and generates results comparable to those obtained via MATLAB, offering a practical alternative for users without access to that software. A step‐by‐step algorithmic overview of the fitting procedure is provided in Supporting Information S2: Notes S3, while explanatory comments are embedded directly with both the MATLAB script and the Excel template.

## 3 Results and Discussion

### 3.1 Bias in Determining Γ* and D_L_ Using Linear Regressions From 5°C to 40°C

**Figure 2:**
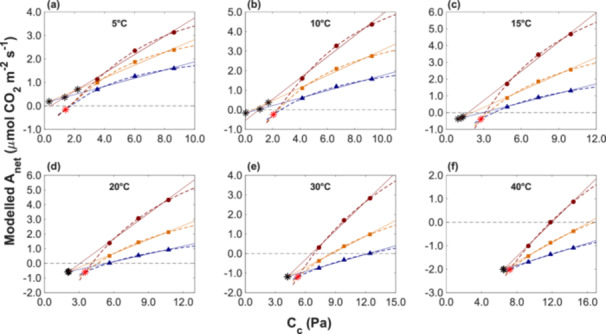
Modelled net assimilation rate as a function of chloroplastic CO_2_ partial pressure (C_c_). Dashed lines represent simulations from the FvCB model, with corresponding solid symbols. Solid lines show linear regressions fitted to these modelled data points. The three light intensities used (300, 150, and 80 µmol photons m^−2^ s^−1^) are indicated by dark red, orange, and blue, respectively. Panels (a) to (f) show simulations at 5°C, 10°C, 15°C, 20°C, 30°C, and 40°C, in the order shown. Black asterisks indicate the intersections of the linear regressions, while the red asterisk marks the prescribed intersection used to simulate the reference values of Γ* and D_L_ at each temperature. [Color figure can be viewed at wileyonlinelibrary.com]

Figure 1 illustrates the erroneous estimates of Γ* and D_L_ based on the A_net_‐C_c_ curve at 6°C using the Laisk method with linear regressions, where the Γ*_exp_ from the linear regression locates beyond the measured range of CO_2_ partial pressures. Our simulations across temperatures from 5°C to 40°C (Figure 2) demonstrated that the estimates obtained via linear regression were highly inaccurate at low temperatures, for example at 5°C and 10°C (Figures 2a,b). At low temperatures, the regression lines often intersected with positive net assimilation rates that would erroneously imply negative values for D_L_. Moreover, the intersections from different regression pairs were scattered and diverged substantially from the Γ*_ref_ and D_L_ values used in the simulations. At higher temperatures, estimates of D_L_ based on the Laisk method became physiologically meaningful. At 15°C (Figure 2c), the regression lines intersected with negative net assimilation rates, although the intersecting points still occurred at C_c_ well below the Γ*_ref_ values used in the simulations. At 20, 30, and 40°C (Figure 2d–f), the regression lines intersected at distinct single points with corresponding meaningful estimates of D_L_. However, the inferred estimates of Γ* continued to underestimate Γ*_ref_.

Notably, at higher temperatures, the Γ* values tended to converge more closely to Γ*_ref_, suggesting that the limitations of the Laisk method became markedly more pronounced under moderate and low temperatures. This bias at lower temperatures could be linked to the substantially lower CO_2_ partial pressure values corresponding to Γ*_exp_, and the more pronounced curvature in the A_net_‐C_c_ relationship.

**Figure 3:**
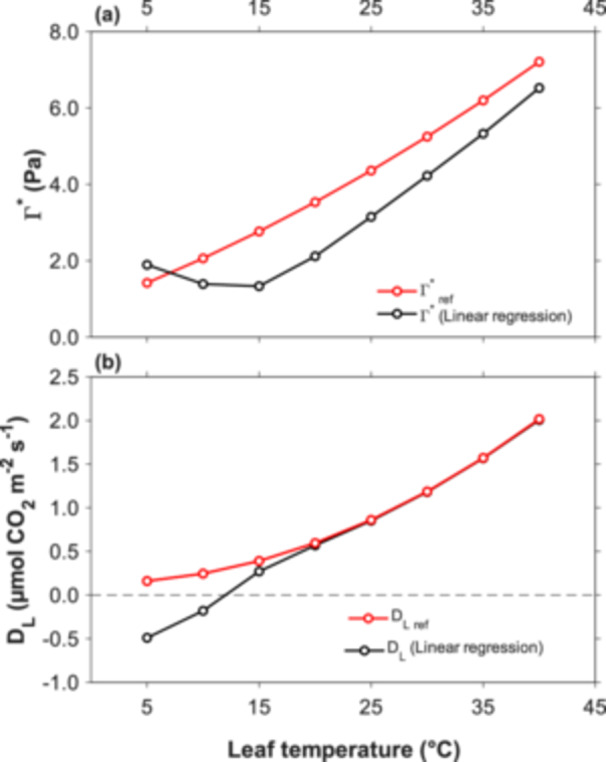
Temperature‐dependent variations in Γ* and D_L_ from 5°C to 40°C. (a) Comparison between the Γ*_ref_ values (solid red line) used in the FvCB model and the Γ* estimates derived from the linear regression intercepts (solid black line). (b) Comparison between the modelled D_l‐ref_ values (solid red line) and the D_L_ estimates from the linear regression intercepts (solid black line). [Color figure can be viewed at wileyonlinelibrary.com]

Figure 3 summarises the values of Γ* and D_L_ estimated using linear regressions (black), in comparison with the reference values used in our simulation based on the FvCB model (red), across a temperature range from 5°C to 40°C. These results are based on the modelled data shown in Figure 2 and reflect the outcomes of estimating Γ* and D_L_ applying linear relationships to the simulated A_net_‐C_c_ curves. Overall, Γ* values obtained from the linear regressions were underestimated at all temperatures except at 5°C (Figure 3a), where Γ* was overestimated. This deviation is explained by the position of the line intersections at 5°C (Figure 2a). The magnitude of these errors was notable. At 15°C, Γ* was underestimated by 1.43 Pa, and even at 40°C, where the issue was less pronounced, the underestimation remained at 0.69 Pa. Regarding D_L_ (Figure 3b), the estimates closely matched the modelled values at temperatures of 20°C and above. However, D_L_ was strongly underestimated below 20°C, producing physiologically meaningless negative values at 5°C and 10°C.

At this point, it is necessary to also draw attention to one of the complications of finding generic patterns. In applying the Laisk method, one typically uses three low‐light intensities that generate different levels of RuBP regeneration and allow the identification of their cross‐over points that define both Γ* and D_L_. If rates are controlled by RuBP regeneration, fitting linear relationships to inherently curved responses consistently leads to mathematical errors, essentially affecting the estimation of Γ*, while D_L_ can still be estimated correctly. This is the pattern seen at higher temperatures in Figure 3.

However, depending on the light levels used and the rates of Rubisco activity at different temperatures, the readings at highest light levels may or may not be limited by Rubisco activity instead of RuBP regeneration. This does not invalidate the use of the Laisk method, but it alters the pattern of errors. In our simulations, as temperature was decreased, Rubisco activity eventually became low enough to become the rate‐limiting step at our highest used light level of 300 µmol photons m^−2^ s^−1^. With our specific parameterisation, this limitation occurred at temperatures of 15°C and below, causing the error introduced by linear relationships to shift from affecting Γ* estimates to affecting D_L_ estimates. These are still equally important estimation errors, but they manifest themselves in different ways (Figure 3).

Our results highlighted the challenge in accurately estimating Γ* and D_L_ using the Laisk method, particularly at low temperatures (Tcherkez and Atkin 2021; Way et al. 2019). This difficulty arises because linear regressions fitted to data at different light intensities do not adequately capture the curvature inherent in the A_net_‐C_c_ relationship (Figure 2). As a result, this can lead to erroneous estimates of D_L_, often resulting in physiologically meaningless values (Atkin et al. 2000; Way et al. 2019). This methodological flaw can be amplified when the measured C_c_ range does not include the Γ*_exp_. In our example illustrated in Figure 1, the C_c_ range, did not include the value of Γ*_exp_. At 6°C, Γ*_exp_ was around 1.54 Pa (Brooks and Farquhar 1985), which was significantly lower than the starting point of 3.6 Pa in the measured C_c_ range. Moreover, considering the wide measured C_c_ range of about 5.6 Pa, that is, from Γ*_exp_ + 2.1 Pa to Γ*_exp_ + 7.2 Pa with varying Γ*_exp_ under different temperatures in Figure 1, substantial errors in the estimation of Γ* and D_L_ are expected by using linear regressions in the Laisk method due to the nonlinear nature of the A_net_‐C_c_ curve based on the FvCB model, especially under lower temperatures (Figure 2a,b).

### 3.2 Sensitivity Analysis of Γ* and D_L_ Across Different C_c_ Range From 5°C to 40°C

**Figure 4:**
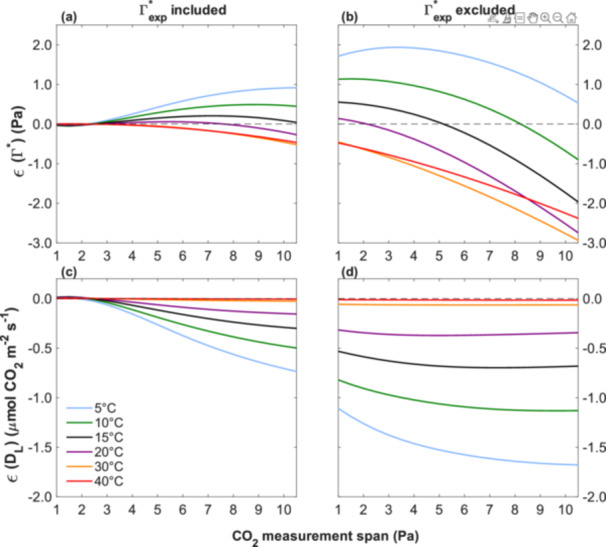
The pattern of Є(Γ*) and Є(D_L_) over the evaluated C_c_ range across temperatures from 5°C to 40°C. Panels (a) and (b) show Є(Γ*) and panels (c) and (d) show Є(D_L_). Panels (a) and (c) correspond to simulations in which Γ*_exp_ was included within the C_c_ range, beginning from 0.5 Pa below Γ*_exp_ with the narrowest range extending to 0.5 Pa above Γ*_exp_. Panels (b) and (d) correspond to simulations in which Γ*_exp_ was excluded, with the C_c_ range begining from 3.0 Pa above Γ*_exp_. In both cases, the upper limit of each subsequent range was increased in 0.5 Pa increments, up to a maximum of 10.5 Pa. [Color figure can be viewed at wileyonlinelibrary.com]

Figures 2 and 3 illustrate the disparities in estimating Γ* and D_L_ using the Laisk method with linear regression, across a C_c_ range that did not include the Γ*_exp_ from the CO_2_ measurement range. As the measured C_c_ range spans from narrow to wide, either including or excluding the Γ*_exp_, the curvature of the A_net_‐C_c_ curve became more apparent, consequently resulting in higher errors in the estimates of Γ* and D_L_. Our simulation (Figure 4) quantifies the estimation errors for Γ*, that is, Є(Γ*), and D_L_,that is, Є(D_L_), across CO_2_ measurement spans from 1 to 10.5 Pa.

When Γ*_exp_ was included in the C_c_ range, Є(Γ*) remained close to zero for spans up to approximately 3 Pa across all temperatures (Figure 4a). Beyond 3 Pa, Є(Γ*) increased at lower temperatures, with errors reaching 0.92 Pa at 5°C and 0.45 Pa at 10°C under the widest C_c_ range tested (10.5 Pa). In contrast, at higher temperatures (30°C and 40°C), Γ* was slightly underestimated, with errors not exceeding −0.5 Pa. Similarly, Є(D_L_) remained approximately zero below the C_c_ range of 3 Pa (Figure 4c) but for spans above 3 Pa, D_L_ was consistently underestimated, that is, Є(D_L_) < 0, across all temperatures. The absolute Є(D_L_) was less than 0.5 µmol CO_2_ m⁻^2^ s⁻^1^ for all temperatures up to a C_c_ range of 7 Pa. At 5°C, underestimation in D_L_ became more pronounced with increasing C_c_ range, reaching −0.7 under the widest range tested. In contrast, at higher temperatures, such as 30°C and 40°C, the underestimation was minimal and remained close to zero throughout.

Substantial overestimations of Γ* were observed at low and moderate temperatures when Γ*_exp_ was excluded from the C_c_ range (Figure 4b), with Є(Γ*) reaching nearly 2 Pa at 5°C under a C_c_ range of 3 Pa. As the C_c_ range increased, the overestimation diminished and eventually turned into underestimation at higher temperatures. For instance, at 40°C, the error dropped from 0.3 Pa at the lowest C_c_ range to −2.5 Pa at the widest C_c_ range (Figure 4b). Figure 4d depicts substantial underestimation of D_L_ at low and moderate temperatures, with the largest discrepancies or Є(D_L_) observed under the widest C_c_ range. At higher temperatures such as 30°C and 40°C, the underestimation remained minimal across the entire C_c_ range.

These results suggest that, to minimise estimation errors using linear regressions in the Laisk method, it is not feasible to rely on C_c_ ranges that exclude Γ*_exp_, as large errors are present even under narrow CO_2_ spans. The absence of Γ*_exp_ leads to systematic bias from the outset, particularly at lower temperatures. When Γ*_exp_ is included, a C_c_ range of up to 3 Pa around it minimises both Є(Γ*) and Є(D_L_), across all temperatures. Including Γ*_exp_ in this range proved essential, particularly at low temperatures, where the risk of over‐ or underestimation was highest. Beyond 3 Pa, errors increased markedly, especially for D_L_ at 5°C.

### 3.3 Estimates of Γ* and D_L_ from Monte Carlo Simulations Using Linear Regressions and the FvCB Model

**Figure 5:**
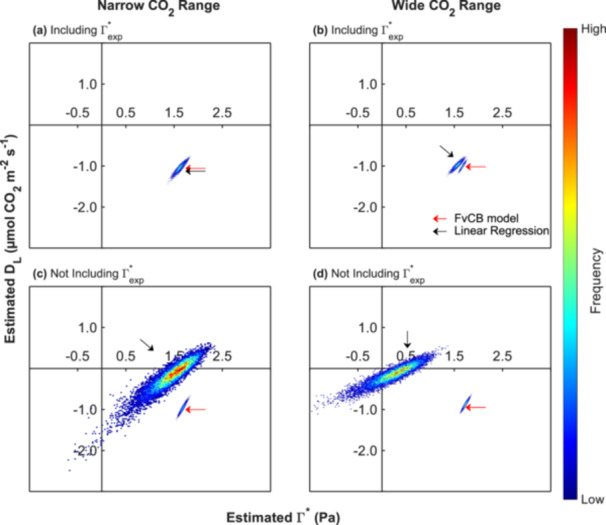
Distribution of intersection points for estimated Γ* and D_L_ from 10,000 Monte Carlo simulations at 6°C. Each panel compares the performance of the FvCB model with Linear Regressions. (a) and (b) refer to analyses that included Γ*_exp_, with (a) using a narrow CO_2_ range and (b) using a wide CO_2_ range. In contrast, the analyses illustrated in panels (c) and (d) did not include Γ*_exp_, with (c) employing a narrow and (d) a wider CO_2_ range. Heat maps display the density of intersection points: warmer colours indicate higher concentrations of points, while cooler colours represent lower densities. Black arrows indicate the dispersion of intersection points obtained using linear regressions, while red arrows represent those obtained using the FvCB model. [Color figure can be viewed at wileyonlinelibrary.com]

Here, we assessed how IRGA measurement errors would affect the estimates of Γ* and D_L_. Figure 4 illustrated that theoretically, better estimates of these parameters could be obtained if one used a narrow CO_2_ measurement range, preferably including the value of Γ*_exp_ when using linear regressions. In practice, that approach would be curtailed by inevitable experimental error in gas‐exchange measurements that require a wider measurement range to partly negate the importance of random measurement errors. We, therefore, conducted a Monte Carlo approach using both the linear regression and the FvCB model, across narrow and wide CO_2_ ranges, and with or without inclusion of Γ*_exp_. For that analysis, we calculated expected A_net_ from simulations of the FvCB model and then randomly modified each point to emulate the effect of experimental error. See the Materials and Methods for further details. The results are summarised in Table 1 and visualised in Figure 5.

**Table 1:** Estimates of Γ* and D_L_ based on 10,000 simulations with varying ranges of CO_2_ partial pressures and two estimation methods. For simulations including Γ*_exp_ in the data set, the CO_2_ ranges were either narrow (1.2–3.6 Pa) or wide (1.2–6.6 Pa). For simulations excluding Γ*_exp_, the narrow range was 3.6–6.6 Pa and the wide range was 3.6–9.2 Pa. Values are presented as mean ± standard deviation. Simulations were conducted for a leaf temperature of 6°C and with assumed true values of 1.67 Pa for Γ* and 1.0 µmol CO_2_ m^−^^2^ s^−^^1^ for D_L_.

Method	Γ*_exp_	CO_2_ range	Γ* (Pa)	D_L_ (µmol CO_2_ m^−2^ s^−1^)
FvCB model	Included	Narrow	1.70 ± 0.04	0.98 ± 0.07
		Wide	1.67 ± 0.03	1.01 ± 0.05
	Excluded	Narrow	1.68 ± 0.04	0.96 ± 0.09
		Wide	1.73 ± 0.04	0.87 ± 0.07
Linear regression	Included	Narrow	1.60 ± 0.06	1.06 ± 0.09
		Wide	1.56 ± 0.06	0.98 ± 0.07
	Excluded	Narrow	1.44 ± 0.36	0.18 ± 0.34
		Wide	0.24 ± 0.37	0.14 ± 0.21

When applying the FvCB model, the estimated values for Γ* and D_L_ were consistent and tightly clustered, regardless of whether Γ*_exp_ was included, or whether the CO_2_ range was narrow or wide (Table 1). In contrast, estimates obtained from linear regression diverged substantially depending on the inclusion of Γ*_exp_. When Γ*_exp_ was included, D_L_ estimates remained close to the true values: 1.06 µmol CO_2_ m^−^^2^ s^−^^1^ for the narrow range and 0.98 ± 0.07 µmol CO_2_ m^−^^2^ s^−^^1^ for the wide range. However, Γ* was still underestimated in both cases. When Γ*_exp_ was excluded, the estimates became highly inaccurate, particularly under wide CO_2_ ranges, where Γ* dropped to 0.24 Pa and D_L_ to 0.14 µmol CO_2_ m^−^^2^ s^−^^1^ (Table 1).

Figure 5 illustrates the distribution of intersection points derived from the simulated experiments using the Monte Carlo method. When the FvCB model was integrated into the Laisk method, the resulting estimates formed compact and consistent clusters across all treatments, demonstrating high robustness to input data variability. Similarly, linear regressions that include Γ*_exp_ yielded distributions more closely aligned with those obtained from the FvCB model, particularly under narrow CO_2_ ranges (Figure 5a). This suggests that, under such conditions, the choice of analysis methods would have little impact on the inferred values of Γ* and D_L._ However, even then, the use of a wide CO_2_ range resulted in a slight underestimation of Γ* (Figure 5b).

Problems with the use of linear regressions for analysis became much worse when the available data did not include Γ*_exp_, and the linear regression approach produced a broader and more dispersed distribution of intersection points. Under these conditions, the linear regression approach was likely to produce erroneous estimates, in extreme cases even including negative values for both Γ* and D_L_ (Figures 5c,d). These conceptual flaws invalidate the derivation of any Γ* and D_L_ estimates with linear analysis if they use only CO_2_ partial pressures that do not include Γ*_exp_.

### 3.4 Estimation of Γ* and D_L_ Using the FvCB Model and Linear Regressions Applied to Experimental Data

**Figure 6:**
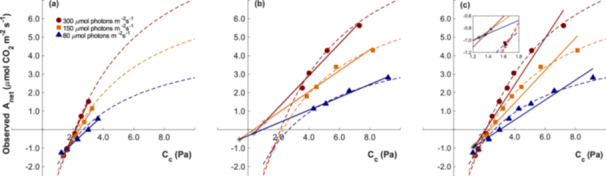
Observed net photosynthetic rate (A_net_) as a function of chloroplastic CO_2_ partial pressure (C_c_) measured at a leaf temperature of 6°C under three light intensities: 300 (dark red), 150 (orange), and 80 (blue) µmol photons m^−2^ s^−1^. Experimental data are shown as individual points. Solid lines represent linear regressions, while dotted lines indicate theoretical fits based on the FvCB model. (a) corresponds to fits derived from the data obtained over a narrow CO_2_ range (1.2–3.6 Pa) that included Γ*_exp_, while (b) shows fits from the data obtained over a wider CO_2_ range (3.6–9.2 Pa) that did not include Γ*_exp_. (c) displays fits derived from the entire experimental CO_2_ range (1.2–9.2 Pa). The inset in (c) provides a magnified view of the region where the regression lines and modelled curves intersect, highlighting the differences between the intersection points generated by the linear regressions and the intersection obtained with the Farquhar model. The red asterisk marks the intersection point of the three A_net_‐C_c_ curves, while black asterisks indicate the intersections of the three linear regressions at each light intensity. [Color figure can be viewed at wileyonlinelibrary.com]

Figure 6 revisits the same experimental data set presented earlier in Figure 1, now reanalysed using both linear regressions and the FvCB model at 6°C. In (a), fits were performed over a narrow CO_2_ range (1.2–3.6 Pa) that included Γ*_exp_. Under these conditions, the intersection points derived from linear regressions (black asterisks) were consistent across light intensities and closely aligned with the intersection point generated by the FvCB model (red asterisk). This alignment highlights the importance of including Γ*_exp_ in measurements for the Laisk method, as it reduces bias and provides physiologically plausible estimates of both Γ* and D_L_, even when linear analyses are used.

In contrast, panel (b) shows the same data set fitted using a broader CO_2_ range (3.6–9.2 Pa) that did not include Γ*_exp_. Here, the intersection points from linear regressions (black asterisks) diverged substantially and led to implausible estimates, including negative values for respiration and unrealistic Γ*. Meanwhile, the FvCB model continued to produce a physiologically consistent fit, with the modelled intersection point remaining robust despite the absence of Γ*_exp_ in the data range. Together, these results reinforce the problematic use of linear regressions for analysis when Γ*_exp_ is excluded. By contrast, use of the FvCB model demonstrated greater robustness and reliability, offering consistent and biologically meaningful estimates even when Γ*_exp_ was not included in the CO_2_ measurement range.

Panel (c) pools the data from panels a and b to compare both approaches across the full C_c_ range (1.2–9.2 Pa). At the main scale the linear regressions and the FvCB fit look similar, yet the inset in the upper right shows a consistent leftward and downward shift of the linear regression intersection relative to the modelled intersection. This shift indicates systematic underestimation of Γ* and D_L_ by the linear regressions compared to the FvCB model.

## 4 General Discussion

### 4.1 Methodological Limitation of the Classical Laisk Approach

Γ* and D_L_ are key parameters for describing photosynthetic CO_2_ responses, and the Laisk method has long been used to estimate them (Berghuijs et al. 2019; Gong et al. 2018). However, our findings showed a fundamental methodological limitation inherent in this approach, because it implicitly assumes linearity in the A_net_‐C_c_ relationship, despite strong theoretical and empirical evidence that photosynthesis exhibits a nonlinear response to CO_2_ (Farquhar et al. 1980; Onoda 2005; Zhang 2010). This linearity assumption introduces a systematic bias, especially when wide CO_2_ ranges are used, as the deviation between linear and nonlinear models increases with the span of CO_2_ partial pressures used (Figure 4). Consequently, use of linear relationships may result in implausible and physiologically unrealistic estimates, such as negative values for Γ* or D_L_ (Figures 1 and 5).

### 4.2 Effects of CO_2_ Range and Inclusion of the Expected Γ*

Negative D_L_ estimates have also been reported in other studies of C_3_ species when linear regressions were applied across wide CO_2_ ranges (Atkin et al. 2000; Kroner and Way 2016; Way et al. 2019). For example, Way et al. (2019) used C_i_ values ranging from 4.0 to 15.0 Pa, a wide range which did not include Γ*_exp_ for the tested temperature. As a result, they obtained negative D_L_ values, indicating positive net CO_2_ uptake, which most likely reflected methodological artefacts caused by not including Γ*_exp_ and the use of wide CO_2_ ranges.

Our theoretical analysis indicated that excluding Γ*_exp_ introduces systematic errors, while its inclusion markedly improves the reliability of estimates obtained through linear regression, as shown in Figure 4. Although Way et al. (2019) did not explicitly aim to test for the effect of inclusion of Γ*_exp_ in their research, when it fell within their measurement range, their estimated D_L_ values were positive. However, even when Γ*_exp_ is included, using wide CO_2_ ranges can still introduce substantial error, as shown in our simulations (Figures 4a,c). For example, Way et al. (2019) reported measurements at 4.0 and 15.0 Pa, hence over a measurement span of 11.0 Pa. Over that range, the FvCB model predicts considerable deviation from linearity in the A_net_‐C_c_ response. Narrower CO_2_ ranges reduce that problem by sampling a more localised and nearly linear segment of the curve, while broader ranges encompass greater curvature, increasing the risk of inaccurate estimates. Implicitly assuming linearity across wide CO_2_ ranges is misleading and results in notable errors in the estimation of both Γ* and D_L_.

These errors are further exacerbated when the selected CO_2_ range does not include Γ*_exp_, as the regression then requires extrapolation beyond the measured data. This issue is especially pronounced at low temperatures where Γ* is lower, the curvature of the A_net_‐C_c_ response is more prominent and owing to instrument limitations, the CO_2_ partial pressures used often lie above Γ*_exp_.

### 4.3 Influence of Temperature and Published Discrepancies

A literature review by Walker and Ort (2015), reported variability in the intercellular CO_2_ photocompensation point at 25°C, with values ranging from 3.1 to 4.9 Pa, representing a spread of 1.8 Pa at this single temperature. As shown in Figure 3, our modelled estimate of Γ* using linear regressions can lead to an underestimate Γ*, with the extent of underestimates depending on the range of partial pressures used and whether it includes or excludes the Γ*_exp_ (Figure 4). This could account for the range of values reported by Walker and Ort (2015).

This variability in the estimates obtained using the Laisk method often arises from the use of CO_2_ ranges that do not include the region around Γ*, especially at lower temperatures where Γ* is significantly lower than at higher temperatures. This then requires extrapolation outside the range of measurement CO_2_ partial pressures and amplifies the problem of non‐capture of the existing curvature in the A_net_‐C_c_ curve. While this may sometimes reflect instrumental constraints, it may also indicate limited awareness about the importance of selecting a range of CO_2_ partial pressures that include Γ*_exp_ to ensure accurate estimation of both parameters.

### 4.4 Uncertainty Analysis via Monte Carlo Simulations

Our Monte Carlo simulations provided additional evidence of the inherent uncertainty in estimating Γ* and D_L_ using linear regression or the FvCB model. We found that even small measurement errors in net CO_2_ assimilation rates that are within the precision range of modern gas exchange analysers can lead to substantial variability in the estimated intersection points of linear regressions (Figure 5). This effect is particularly pronounced when the selected CO_2_ range does not include Γ*_exp_, resulting in a wide spread of possible outcomes and, in some cases, physiologically implausible estimates such as negative values for Γ* and D_L_ (Figure 5).

Even when Γ*_exp_ is included within the measurement range, applying linear regressions across a wide CO_2_ range still leads to a slight underestimation of Γ* (Table 1, Figure 5b). In contrast, when a narrow CO_2_ range is used, the intersection points obtained through both linear regression and the FvCB model converge to nearly identical values (Figure 5a). This suggests that under these specific conditions, both methods can yield comparable estimates of Γ* and D_L_. The problem of estimating the nonlinear A_net_‐C_c_ curve with linear relationships could, in principle, be ameliorated by using a narrow range of CO_2_ partial pressures. A limited range of C_c_ may locally approximate linearity within the A_net_‐C_c_ curve, which would improve the validity of using linear regression in such cases.

When both approaches were tested under the same level of experimental error, the Monte Carlo analysis showed that the variation in Γ* and D_L_ estimates was markedly lower when using the FvCB model. Considering the physiological and technical constraints discussed above, as well as the findings presented in Figure 5 and Table 1, implementing the FvCB model in the Laisk Method offers a practical and robust approach for estimating Γ* and D_L_ across a range of temperatures. The FvCB model performs reliably across both wide and narrow CO_2_ ranges and effectively avoids the systematic errors associated with fitting linear regressions and consistently outperforms it, even when Γ*_exp_ is not included in the CO_2_ measurement range.

### 4.5 Advantages of Integrating the FvCB Model Into the Laisk Method

To demonstrate the use and application of our methodological proposal to incorporate photosynthetic theory into the Laisk method, we analysed the A_net_‐C_i_ data set of Yin et al. (2011) and Yin and Amthor (2024). In their article, Yin and Amthor (2024) calculated the apparent Γ* (C_i_*) from oxygen partial pressure and Rubisco specificity, resulting in 35 µmol mol^−1^, and then fixed this value to determine the apparent D_L_. However, the original Laisk method allows both parameters to be estimated from the intersection of at least three linear regressions constructed from A_net_‐C_i_ curves measured at three sub‐saturating light intensities under low CO_2_. Fixing C_i_* in the Laisk method can introduce methodological error because it constrains the intersection to a value of C_i_* set a priori rather than letting the data determine it, affecting the estimation of D_L_.

This is inconsistent with the Laisk method and can bias D_L_, especially when the analysed C_i_ range is wide or not close to Γ*_exp_. Yin and Amthor (2024) reported a D_L_ value of 0.97 µmol m^−2^ s^−1^ using the linear method, and 1.21 µmol m^−2^ s^−1^ using the non‐rectangular hyperbolic equation they proposed, based on chlorophyll *a* fluorescence data. However, when we reanalysed their data, we found that the linear regressions yielded three intersections in markedly different locations and therefore widely divergent estimates of C_i_* and D_L_, as shown in Supporting Information S2: Figure S3a. This divergence arose because we allowed the data to determine the intersection points rather than imposing a fixed C_i_*. The intersections were so widely separated that averaging them to estimate C_i_* and D_L_ was inappropriate.

We then analysed the same data with our proposed approach (See Supporting Information S2: Figure S3b). We used the J_max_ and θ values reported by Yin and Amthor (2024), requiring only three parameters to be fitted in our model: C_i_*, D_L_ and V_c__max_. This yielded estimated values of C_i_* = 41.6 µmol mol^−1^ and D_L_ = 0.57 µmol m^−2^ s^−1^, which were substantially different from those reported by Yin and Amthor (2024).

When we restricted the analysis to a narrower CO_2_ range, as recommended in our methodology for more reliable linear regression estimates (Supporting Information S2: Figure S3c), the three intersections from the linear regressions lay closer together, yielding a C_i_* much nearer to that from our model. Running the model on the same narrow range (dotted line Supporting Information S2: Figure S3c) gave values that were very close to those from the wide range (Supporting Information S2: Figure S3b). C_i_* changes from 41.6 to 42.0 µmol mol^−1^ and D_L_ from 0.57 to 0.64 µmol m^−2^ s^−1^.

Despite the apparent proximity of the intersections between the linear regressions and the FvCB model when a narrower CO_2_ range was used (Supporting Information S2: Figure S3c), C_i_* was still underestimated by 5.7% and D_L_ by 23.4%. It showed that even at moderate temperatures, where the issue is less pronounced than at low temperatures, linear regressions can still yield misleading estimates unless a narrow CO_2_ range is used that includes or closely brackets Γ*_exp_ (Supporting Information S2: Figure S3a). We, therefore, recommend using our approach based on the FvCB model to obtain more accurate estimates of Γ* and D_L_.

### 4.6 Conclusion and Perspective

Our study shows that the classical Laisk method, when applied across wide CO_2_ ranges or without bracketing Γ*_exp_, can yield biased or physiologically implausible estimates of Γ* and D_L_. Integrating the Farquhar, von Caemmerer, and Berry (FvCB) photosynthesis model into the Laisk method:

- Reduces systematic bias by capturing the inherent curvature of the A_net_‐C_c_ relationship.
- Maintains accuracy across a wide spectrum of temperatures and CO_2_ partial pressures.
- Lowers the variance of Γ* and D_L_ estimates under realistic measurement noise, as shown by Monte Carlos analysis.

Refining the Laisk approach with a mechanistic framework not only improves parameter estimation in C_3_ plants but also enhances the reliability of carbon‐flux models used to predict plant responses to climate change.

For future studies, we recommend determining Γ*_exp_ for each target temperature, based on prior experimental work, and selecting CO_2_ ranges that bracket it. Measurements that include or closely approach Γ* are likely to yield more accurate and interpretable estimates of Γ* and D_L_. In addition, we recommend assessing potential changes in D_L_ with variation in light and CO_2_, both during the application of the Laisk method and under natural conditions (e.g. cloud cover, photoperiod, ambient CO_2_). Extending this approach across species, functional types and biomes, and across stress regimes will strengthen the understanding of light‐driven CO_2_ release and improve its representation in mechanistic carbon‐exchange components of climate models, with implications for crop resilience and management and for improved understanding of ecosystem functioning and ecosystem services.

## Conflicts of Interest

The authors declare no conflicts of interest.

## Supporting information

Supplementary File Excel Template.

Supplementary Material.

## Data Availability

All data supporting the findings of this study are available within the article and its Supporting Information S2: Supporting Material. This includes the raw data used for parameter estimation, a supporting Excel template, and a MATLAB script developed to estimate Γ* and D_L_ by applying photosynthetic theory within the framework of the Laisk method.

## References

[pce70195-bib-0001] Abadie, C. , J. Lalande , C. Dourmap , A. M. Limami , and G. Tcherkez . 2024. “Leaf Day Respiration Involves Multiple Carbon Sources and Depends on Previous Dark Metabolism.” Plant Cell & Environment 47: 2146–2162. 10.1111/pce.14871 38444114

[pce70195-bib-0002] Amthor, J. S. , G. W. Koch , J. R. Willms , and D. B. Layzell . 2001. “Leaf O2 Uptake in the Dark Is Independent of Coincident CO2 Partial Pressure.” Journal of Experimental Botany 52: 2235–2238. 11604463 10.1093/jexbot/52.364.2235

[pce70195-bib-0003] Arcus, V. L. , E. J. Prentice , J. K. Hobbs , et al. 2016. “On the Temperature Dependence of Enzyme‐Catalyzed Rates.” Biochemistry 55: 1681–1688. 26881922 10.1021/acs.biochem.5b01094

[pce70195-bib-0004] Atkin, O. K. , J. R. Evans , M. C. Ball , H. Lambers , and T. L. Pons . 2000. “Leaf Respiration of Snow Gum in the Light and Dark. Interactions Between Temperature and Irradiance.” Plant Physiology 122: 915–924. 10712556 10.1104/pp.122.3.915 PMC58928

[pce70195-bib-0006] Berghuijs, H. N. C. , X. Yin , Q. T. Ho , M. A. Retta , B. M. Nicolaï , and P. C. Struik . 2019. “Using a Reaction‐Diffusion Model to Estimate Day Respiration and Reassimilation of (Photo) Respired Co_2_ in Leaves.” New Phytologist 223: 619–631. 31002400 10.1111/nph.15857 PMC6618012

[pce70195-bib-0007] Bernacchi, C. J. , A. R. Portis , H. Nakano , S. von Caemmerer , and S. P. Long . 2002. “Temperature Response of Mesophyll Conductance. Implications for the Determination of Rubisco Enzyme Kinetics and for Limitations to Photosynthesis In Vivo.” Plant Physiology 130: 1992–1998. 12481082 10.1104/pp.008250 PMC166710

[pce70195-bib-0008] Brooks, A. , and G. D. Farquhar . 1985. “Effect of Temperature on the Co_2_/O_2_ Specificity of Ribulose‐1,5‐bisphosphate Carboxylase/Oxygenase and the Rate of Respiration in the Light. Estimates From Gas‐Exchange Measurements on Spinach.” Planta 165: 397–406. 24241146 10.1007/BF00392238

[pce70195-bib-0009] Buckley, T. N. , and G. D. Farquhar . 2004. “A New Analytical Model for Whole‐Leaf Potential Electron Transport Rate.” Plant, Cell & Environment 27: 1487–1502.

[pce70195-bib-0010] Bush, F. A. , E. A. Ainsworth , A. Amtmann , et al. 2024. “A Guide to Photosynthetic Gas Exchange Measurements: Fundamental Principles, Best Practice and Potential Pitfalls.” Plant, Cell & Environment 47: 3344–3364. 10.1111/pce.14815 38321805

[pce70195-bib-0011] Farquhar, G. D. , and F. A. Busch . 2017. “Changes in the Chloroplastic Co_2_ Concentration Explain Much of the Observed Kok Effect: A Model.” New Phytologist 214: 570–584. 28318033 10.1111/nph.14512

[pce70195-bib-0012] Farquhar, G. D. , S. von Caemmerer , and J. A. Berry . 1980. “A Biochemical Model of Photosynthetic CO_2_ Assimilation in Leaves of C_3_ Species.” Planta 149: 78–90. 24306196 10.1007/BF00386231

[pce70195-bib-0013] Flexas, J. , A. Diaz‐Espejo , J. Galmés , R. Kaldenhoff , H. Medrano , and M. Ribas‐Carbo . 2007. “Rapid Variations of Mesophyll Conductance in Response to Changes in Co_2_ Concentration Around Leaves.” Plant, Cell & Environment 30: 1284–1298. 10.1111/j.1365-3040.2007.01700.x 17727418

[pce70195-bib-0014] Gong, X. Y. , G. Tcherkez , J. Wenig , R. Schäufele , and H. Schnyder . 2018. “Determination of Leaf Respiration in the Light: Comparison Between an Isotopic Disequilibrium Method and the Laisk Method.” New Phytologist 218: 1371–1382. 29611899 10.1111/nph.15126

[pce70195-bib-0015] Herrmann, H. A. , J. M. Schwartz , and G. N. Johnson . 2020. “From Empirical to Theoretical Models of Light Response Curves—Linking Photosynthetic and Metabolic Acclimation.” Photosynthesis Research 145: 5–14. 31654195 10.1007/s11120-019-00681-2 PMC7308256

[pce70195-bib-0016] Heskel, M. A. 2018. “Small Flux, Global Impact: Integrating the Nuances of Leaf Mitochondrial Respiration in Estimates of Ecosystem Carbon Exchange.” American Journal of Botany 105: 815–818. 29807386 10.1002/ajb2.1079

[pce70195-bib-0017] Huntingford, C. , O. K. Atkin , A. Martinez‐De La Torre , et al. 2017. “Implications of Improved Representations of Plant Respiration in a Changing Climate.” Nature Communications 8: 1602. 10.1038/s41467-017-01774-z PMC5693865 29150610

[pce70195-bib-0018] Kirschbaum, M. U. F. , and G. Farquhar . 1984. “Temperature Dependence of Whole‐Leaf Photosynthesis in *Eucalyptus pauciflora* Sieb. ex Spreng.” Functional Plant Biology 11: 519–538.

[pce70195-bib-0019] Kirschbaum, M. U. F. , and G. D. Farquhar . 1987. “Investigation of the CO_2_ Dependence of Quantum Yield and Respiration in *Eucalyptus pauciflora* .” Plant Physiology 83: 1032–1036. 16665319 10.1104/pp.83.4.1032 PMC1056496

[pce70195-bib-0020] Kitao, M. , E. Agathokleous , H. Harayama , K. Yazaki , and H. Tobita . 2021. “Constant Ratio of C_c_ to C_i_ Under Various CO_2_ Concentrations and Light Intensities, and During Progressive Drought, in Seedlings of Japanese White Birch.” Photosynthesis Research 147: 27–37. 33068256 10.1007/s11120-020-00788-x

[pce70195-bib-0021] Kroner, Y. , and D. A. Way . 2016. “Carbon Fluxes Acclimate More Strongly to Elevated Growth Temperatures Than to Elevated CO_2_ Concentrations in a Northern Conifer.” Global Change Biology 22: 2913–2928. 26728638 10.1111/gcb.13215

[pce70195-bib-0022] Laisk, A. 1977. Kinetics of Photosynthesis and Photorespiration in C_3_ Plants. Nauka.

[pce70195-bib-0023] Li, Y. , X. Song , S. Li , W. T. Salter , and M. M. Barbour . 2020. “The Role of Leaf Water Potential in the Temperature Response of Mesophyll Conductance.” New Phytologist 225: 1193–1205. 31545519 10.1111/nph.16214

[pce70195-bib-0024] Liang, L. L. , V. L. Arcus , M. A. Heskel , et al. 2018. “Macromolecular Rate Theory (MMRT) Provides a Thermodynamics Rationale to Underpin the Convergent Temperature Response in Plant Leaf Respiration.” Global Change Biology 24: 1538–1547. 29030907 10.1111/gcb.13936

[pce70195-bib-0026] Machino, S. , S. Nagano , and K. Hikosaka . 2021. “The Latitudinal and Altitudinal Variations in the Biochemical Mechanisms of Temperature Dependence of Photosynthesis Within *Fallopia japonica* .” Environmental and Experimental Botany 181: 104248.

[pce70195-bib-0028] Needham, J. F. , S. Dey , C. D. Koven , et al. 2025. “Vertical Canopy Gradients of Respiration Drive Plant Carbon Budgets and Leaf Area Index.” New Phytologist 246: 144–157. 39972995 10.1111/nph.20423 PMC11883058

[pce70195-bib-0029] Onoda, Y. 2005. “Seasonal Change in the Balance Between Capacities of RuBP Carboxylation and RuBP Regeneration Affects CO_2_ Response of Photosynthesis in *Polygonum cuspidatum* .” Journal of Experimental Botany 56: 755–763. 15596479 10.1093/jxb/eri052

[pce70195-bib-0030] Peisker, M. , and H. Apel . 2001. “Inhibition by Light of CO_2_ Evolution From Dark Respiration: Comparison of Two Gas Exchange Methods.” Photosynthesis Research 70: 291–298. 16252174 10.1023/A:1014799118368

[pce70195-bib-0032] Ranathunga, K. N. , J. Evans , N. Toth , S. Brown , L. L. Van Eerd , and C. Wagner‐Riddle . 2025. “Net Ecosystem Carbon Budget and Net Greenhouse Gas Emissions Under Diverse Crop Rotation Using Cover Crops Compared to a Conventional Crop Rotation.” Agriculture, Ecosystems & Environment 381: 109418.

[pce70195-bib-0033] Schmiege, S. C. , T. D. Sharkey , B. Walker , J. Hammer , and D. A. Way . 2023. “Laisk Measurements in the Non‐Steady‐State: Tests in Plants Exposed to Warming and Variable CO_2_ Concentrations.” Plant Physiology 193: 1045–1057. 37232396 10.1093/plphys/kiad305 PMC10517191

[pce70195-bib-0034] Scott, D. W. 2011. “Box‐Muller Transformation.” WIREs Computational Statistics 3: 177–179.

[pce70195-bib-0035] Shrestha, A. , X. Song , and M. M. Barbour . 2019. “The Temperature Response of Mesophyll Conductance, and Its Component Conductances, Varies Between Species and Genotypes.” Photosynthesis Research 141: 65–82. 30771063 10.1007/s11120-019-00622-z

[pce70195-bib-0036] Smith, N. G. , and J. S. Dukes . 2013. “Plant Respiration and Photosynthesis in Global‐Scale Models: Incorporating Acclimation to Temperature and CO_2_ .” Global Change Biology 19: 45–63. 23504720 10.1111/j.1365-2486.2012.02797.x

[pce70195-bib-0037] Sun, J. , D. Guan , J. Wu , et al. 2015. “Day and Night Respiration of Three Tree Species in a Temperate Forest of Northeastern China.” iForest ‐ Biogeosciences and Forestry 8: 25–32.

[pce70195-bib-0038] Tcherkez, G. , C. Abadie , C. Dourmap , J. Lalande , and A. M. Limami . 2024. “Leaf Day Respiration: More Than Just Catabolic CO_2_ Production in the Light.” Plant Cell & Environment 47: 2631–2639. 10.1111/pce.14904 38528759

[pce70195-bib-0039] Tcherkez, G. , and O. K. Atkin . 2021. “Unravelling Mechanisms and Impacts of Day Respiration in Plant Leaves: An Introduction to a Virtual Issue.” New Phytologist 230: 5–10. 33650185 10.1111/nph.17164

[pce70195-bib-0040] Tcherkez, G. , P. Gauthier , T. N. Buckley , et al. 2017. “Leaf Day Respiration: Low CO_2_ Flux but High Significance for Metabolism and Carbon Balance.” New Phytologist 216: 986–1001. 28967668 10.1111/nph.14816

[pce70195-bib-0041] Tholen, D. , G. Ethier , B. Genty , S. Pepin , and X. G. Zhu . 2012. “Variable Mesophyll Conductance Revisited: Theoretical Background and Experimental Implications.” Plant, Cell & Environment 35: 2087–2103. 10.1111/j.1365-3040.2012.02538.x 22590996

[pce70195-bib-0042] Ubierna, N. , L. A. Cernusak , M. Holloway‐Phillips , F. A. Busch , A. B. Cousins , and G. D. Farquhar . 2019. “Critical Review: Incorporating the Arrangement of Mitochondria and Chloroplasts Into Models of Photosynthesis and Carbon Isotope Discrimination.” Photosynthesis Research 141: 5–31. 30955143 10.1007/s11120-019-00635-8

[pce70195-bib-0043] Walker, B. J. , and A. B. Cousins . 2013. “Influence of Temperature on Measurements of the CO_2_ Compensation Point: Differences Between the Laisk and O_2_‐Exchange Methods.” Journal of Experimental Botany 64: 1893–1905. 23630324 10.1093/jxb/ert058 PMC3638825

[pce70195-bib-0044] Walker, B. J. , D. J. Orr , E. Carmo‐Silva , M. A. J. Parry , C. J. Bernacchi , and D. R. Ort . 2017. “Uncertainty in Measurements of the Photorespiratory CO_2_ Compensation Point and Its Impact on Models of Leaf Photosynthesis.” Photosynthesis Research 132: 245–255. 28382593 10.1007/s11120-017-0369-8 PMC5443873

[pce70195-bib-0045] Walker, B. J. , and D. R. Ort . 2015. “Improved Method for Measuring the Apparent CO_2_ Photocompensation Point Resolves the Impact of Multiple Internal Conductances to CO_2_ to Net Gas Exchange.” Plant Cell & Environment 38: 2462–2474. 10.1111/pce.12562 25929271

[pce70195-bib-0046] Way, D. A. , M. J. Aspinwall , J. E. Drake , et al. 2019. “Responses of Respiration in the Light to Warming in Field‐Grown Trees: A Comparison of the Thermal Sensitivity of the Kok and Laisk Methods.” New Phytologist 222: 132–143. 30372524 10.1111/nph.15566

[pce70195-bib-0047] Yin, X. , and J. S. Amthor . 2024. “Estimating Leaf Day Respiration From Conventional Gas Exchange Measurements.” New Phytologist 241: 52–58. 37858976 10.1111/nph.19330

[pce70195-bib-0048] Yin, X. , Z. Sun , P. C. Struik , and J. Gu . 2011. “Evaluating a New Method to Estimate the Rate of Leaf Respiration in the Light by Analysis of Combined Gas Exchange and Chlorophyll Fluorescence Measurements.” Journal of Experimental Botany 62: 3489–3499. 21382918 10.1093/jxb/err038 PMC3130174

[pce70195-bib-0049] Zhang, S. 2010. “Temperature Acclimation of Photosynthesis in *Meconopsis horridula* var. Racemosa Prain.” Botanical Studies 51: 457–464.

