# Supplementary material for: Estimating the Photorespiratory CO2 Compensation Point and CO2 Release in the Light Using the Laisk Method Combined With Photosynthetic Theory
Source: Plant Cell Environ. 2025 Sep 23;49(1):80–93. doi: 10.1111/pce.70195 (PMC12675974; doi:10.1111/pce.70195)
Supplement: Supplementary file 2 — Supplementary Material. [file PCE-49-80-s001.docx]

***Supplementary material***

Moreno-Echeverry, Darwin L., Kirshbaum, Miko U.F., Barbour, Margaret M. & Liáng, Lìyǐn L.

Estimating the Photorespiratory CO_2_ Compensation Point and CO_2_ Release in the Light Using the Laisk Method Combined with Photosynthetic Theory

**Contents:**

**Supplementary Table S1.** List of parameters and their values used in the simulations on the FvCB model.

**Supplementary Notes S1.** Procedure for Calculating Temperature Dependency of Parameters in the FvCB Model

**Supplementary Notes S2.** MATLAB script

**Supplementary Notes S3.** Stepwise Procedure for Parameter Fitting and Estimation of Γ* and D_L_ Using the Laisk Method through the FvCB Model.

**Supplementary Figure S1.** Response of mesophyll conductance (gₘ) to environmental conditions

**Supplementary Figure S2.** Sensitivity analysis of the estimated parameters Γ* and D_L_ to mesophyll conductance (gₘ)

**Supplementary Figure S3.** Replot of the Yin et al. 2011, wheat A–C_i_ dataset at 25 °C

**Supplementary Table S1.** List of parameters and their values used in the simulations on the FvCB model.

| Parameter (unit) | Value | Meaning and source. |
| --- | --- | --- |
| e (-) | 0.1 | Empirical factor which transforms the function of Vc into a smooth hyperbola. Kirschbaum and Farquhar (1984). |
| E_a(Kc)_ (*k*J mol⁻¹) | 59.4 | Activation energy for Kc.  Sharkey et al. (2007). |
| E_a(Ko)_ (*k*J mol⁻¹) | 35.9 | Activation energy for Ko.  Sharkey et al. (2007). |
| K_a_ (Pa) | 1.0 | Michaelis-Menten constant for activation of Rubisco by CO_2_ at a given Mg^2+^ concentration and pH.  Kirschbaum and Farquhar (1984). |
| K_c_ 25°C (Pa) | 36.9 | Michaelis-Menten constant for CO_2_.  von Caemmerer and Quick (2000). Jordan and Ogren (1984). |
| K_o_ 25°C (*k*Pa) | 35.1 | Michaelis-Menten constant for O_2_.  von Caemmerer and Quick (2000). Jordan and Ogren (1984). |
| O_2_ (*k*Pa) | 21.0 | Partial pressure of oxygen. |
| P (*k*Pa) | 102.1 | Average pressure in the gas exchange chamber. |
| R (J mol⁻¹ K⁻¹) | 8.314 | Ideal gas constant. |
| α (mol e- mol photons^-1^) | 0.25 | Quantum yield of electron transport. The literature reports values between 0.01 - 0.5 moles m^-2^ s^-1^. |
| θ (-) | 0.30 | Describes the curvature of photosynthesis. The literature reports values between 0.01 - 10 moles m^-2^ s^-1^. |

**Supplementary Notes S1.** Procedure for Calculating Temperature Dependency of Parameters in the FvCB Model

**1. Γ*_ref –_ Temperature dependence**

The temperature dependence of Γ*_ref_ was incorporated based on the empirical function reported by Brooks and Farquhar (1985), originally expressed in μmol mol^-1^. To ensure consistency with the pressure-based units used throughout this study, values were converted to pascals (Pa) by multiplying by the atmospheric pressure (P = 102100 Pa) and dividing by 10^6^. The resulting expression was:

$$\Gamma_{ref}^{*}= \frac{\left[ 0.012\left( T-25 \right)^{2}+1.68\left( T-25 \right)+42.7 \right] \times P}{{10}^{6}}$$

where T is the leaf temperature in °C. This equation captures the response of Γ* to temperature and was used to compute a reference value for Γ* at each temperature point in the model.

**2. D_L ref_ – Temperature dependence**

The temperature dependence of D_L_ was derived empirically from our experimental observations on sunflower leaves. The fitted polynomial regression, valid for the temperature range tested (5–40°C), was:

$$D_{L-ref}= 0.0012\left( T-25 \right)^{2}+0.059\left( T-25 \right)+0.86$$

**3. *V*_cmax_ and *J*_max_ – Temperature dependence using MMRT**

For both *V*_cmax_ and *J*_max_, temperature dependencies were described using the Macromolecular Rate Theory (MMRT), a thermodynamically grounded framework that better captures enzymatic kinetics over wide temperature ranges (Arcus et al., 2016; Liang et al., 2018). MMRT explicitly considers the change in heat capacity (ΔC_p_^‡^) between the ground and transition states of enzyme-catalysed reactions, providing mechanistic insights into temperature responses:

$$\ln\left( k \right)=ln\left( \frac{k_{B}T}{h} \right)-\frac{\Delta H_{T_{0}+}^{\ddagger}\Delta C_{p}^{\ddagger}\left( T-T_{0} \right)}{RT}+ \frac{\Delta S_{T_{0}+}^{\ddagger}\Delta C_{p}^{\ddagger}(\ln T-lnT_{0})}{R}$$

where *k* is the catalytic rate, k_B_ is Boltzmann’s constant, h is Planck’s constant, R is the universal gas constant, ∆H^‡^_T0_ is the change in enthalpy at a reference temperature *T_0_*, ∆S^‡^_T0_ is the change in entropy at *T_0_* and, ∆C^‡^_p_ is the change in heat capacity between the ground and transition states.

For *V*_cmax_, an additional adjustment was made to incorporate thermal deactivation at hight temperatures using the denaturation function based on Peterson et al (2004). This function models the reversible equilibrium between active (*E*_act_) and inactive (*E*_inact_) enzyme forms (K_eq_) and its temperature dependence is described as:

$$\ln\left( K_{eq} \right)= \frac{\Delta H_{eq}}{R} \left( \frac{1}{T_{eq}}-\frac{1}{T} \right)$$

Where:ΔH_eq_ is the enthalpy change associated with the active-inactive enzyme transition and *T_eq_* is the temperature at which the concentration of *E*_act_ and *E*_inact_ are equal. MMRT with deactivation fits five free parameters: the three MMRT parameters ΔH^‡^T_0_, ΔS^‡^T_0_ and ΔCp^‡^, and the two deactivation parameters ΔH_eq_ and T_eq_. Physical constants (kB, h, R) are fixed. All five free parameters were estimated by non-linear least squares fits to *V*_cmax_(T) data.

**Supplementary Notes S2.** MATLAB Script

**Note:** This document contains the original MATLAB code, now formatted in Word as requested.

% Script 1: FvCB Model Parameter Fitting (Generic Version)

% Load experimental data: replace 'YourFile.xlsx' with the name of your data file

[num, txt, raw] = xlsread('YourFile.xlsx'); % <-- Insert your filename here

% Assign columns: ensure the order matches your data (e.g., PFD in column 1, Cc in column 2, A in column 3)

PFD = num(:,1); % Photosynthetic photon flux density

Cc = num(:,2); % Chloroplastic COâ‚‚ partial pressure

A = num(:,3); % Net photosynthesis rate

% Generate a Cc range for model predictions (adjust limits as needed)

Cc_range = InsertRangeHere;

% Fixed environmental and model parameters, replace with your experimental conditions

R = InsertValueHere; % Gas constant (J/mol.Kelvin)

O2 = InsertValueHere; % Oxygen concentration (Pa)

e = InsertValueHere; % Empirical factor (co-limitation)

T = InsertValueHere; % Temperature in °C

Ka = InsertValueHere; % Michaelis-Menten constant for Rubisco activation by CO2

% Optimisation settings and initial parameter guesses

options = optimset('Display', 'off', 'MaxFunEvals', 10000, 'MaxIter', 10000);

initial_guess = [GammaStar_init, DL_init, Vcmax_init, Jmax_init, alpha_init, theta_init]; % <-- Insert initial values

% Fit the model

[temp, fval, exitflag] = fminsearch(@(para) FvCB_Estimation(para, [Cc, PFD], A, R, O2, e, T, Ka), initial_guess, options);

% Check optimisation status

if exitflag <= 0

warning('Optimisation did not converge.');

end

% Display estimated parameters

fprintf('Estimated Gamma*: %.4f\n', temp(1));

fprintf('Estimated DL: %.4f\n', temp(2));

fprintf('Estimated Vcmax: %.4f\n', temp(3));

fprintf('Estimated Jmax: %.4f\n', temp(4));

fprintf('Estimated alpha: %.4f\n', temp(5));

fprintf('Estimated theta: %.4f\n', temp(6));

% Plot data and model fit

figure;

unique_PFD = unique(PFD);

hold on;

% Define colour scheme (adjust as necessary)

colours = lines(length(unique_PFD));

for i = 1:length(unique_PFD)

idx = PFD == unique_PFD(i);

plot(Cc(idx), A(idx), 'o', 'LineWidth', 2, 'MarkerSize', 8, 'Color', colours(i,:), 'MarkerFaceColor', colours(i,:));

end

for i = 1:length(unique_PFD)

A_pred = zeros(size(Cc_range));

for j = 1:length(Cc_range)

A_pred(j) = FvCB_Photosynthesis(temp, Cc_range(j), unique_PFD(i), R, O2, e, T, Ka);

end

plot(Cc_range, A_pred, '--', 'Color', colours(i,:), 'LineWidth', 1.5);

end

xlabel('C_c (Pa)', 'FontSize', 12);

ylabel('A (\mumol CO_{2} m^-^2 s^{-1})', 'FontSize', 12);

title('Model Fit Using the FvCB Model');

hold off;

% --- Supporting Functions ---

function error = FvCB_Estimation(para, data, A_obs, R, O2, e, T, Ka)

Cc = data(:, 1);

PFD = data(:, 2);

Gstar = para(1);

DL = para(2);

Vcmax = para(3);

Jmax = para(4);

alpha = para(5);

theta = para(6);

% Biochemical constants

Kc25 = InsertValueHere; % Michaelis-Menten constant for CO2 at 25°C (Pa)

Ko25 = InsertValueHere; % Michaelis-Menten constant for O2 at 25°C (Pa)

Ea_Kc = InsertValueHere; % Activation energy for Kc (J/mol)

Ea_Ko = InsertValueHere; % Activation energy for Ko (J/mol)

% Temperature adjustment of kinetic constants

Kc = Kc25 * exp(-Ea_Kc / R * (1 / (T + 273.15) - 1 / 298.15));

Ko = Ko25 * exp(-Ea_Ko / R * (1 / (T + 273.15) - 1 / 298.15));

Km = Kc * (1 + O2 / Ko);

A_pred = zeros(size(Cc));

for i = 1:length(Cc)

Vcmax_prime = (Vcmax * Cc(i)) / (Cc(i) + Ka);

Wc = (Vcmax_prime * Cc(i)) / (Cc(i) + Km);

J = (alpha * PFD(i) + Jmax - sqrt((alpha * PFD(i) + Jmax)^2 - 4 * theta * alpha * PFD(i) * Jmax)) / (2 * theta);

Wj = (J * Cc(i)) / (4 * (Cc(i) + 2 * Gstar));

Vc = (Wc + Wj + e - sqrt((Wc + Wj + e)^2 - 4 * Wc * Wj)) / 2;

A_pred(i) = Vc * (1 - Gstar / Cc(i)) + DL;

end

error = mean((A_pred - A_obs).^2);

end

function A = FvCB_Photosynthesis(para, Cc, PFD, R, O2, e, T, Ka)

Gstar = para(1);

DL = para(2);

Vcmax = para(3);

Jmax = para(4);

alpha = para(5);

theta = para(6);

Kc25 = InsertValueHere; % Michaelis-Menten constant for CO2 at 25°C (Pa)

Ko25 = InsertValueHere; % Michaelis-Menten constant for O2 at 25°C (Pa)

Ea_Kc = InsertValueHere; % Activation energy for Kc (J/mol)

Ea_Ko = InsertValueHere; % Activation energy for Ko (J/mol)

Kc = Kc25 * exp(-Ea_Kc / R * (1 / (T + 273.15) - 1 / 298.15));

Ko = Ko25 * exp(-Ea_Ko / R * (1 / (T + 273.15) - 1 / 298.15));

Km = Kc * (1 + O2 / Ko);

Vcmax_prime = (Vcmax * Cc) / (Cc + Ka);

Wc = (Vcmax_prime * Cc) / (Cc + Km);

J = (alpha * PFD + Jmax - sqrt((alpha * PFD + Jmax)^2 - 4 * theta * alpha * PFD * Jmax)) / (2 * theta);

Wj = (J * Cc) / (4 * (Cc + 2 * Gstar));

Vc = (Wc + Wj + e - sqrt((Wc + Wj + e)^2 - 4 * Wc * Wj)) / 2;

A = Vc * (1 - Gstar / Cc) + DL;

end

%% Script 2: Comparison and Visualisation of the FvCB Model and Linear Regression (Generic Version)

% This script compares simulated photosynthesis curves from the FvCB model with experimental data and estimates Gamma* and DL using linear regressions among different light intensities.

% Load experimental data: replace with your file name

[num, txt, raw] = xlsread('YourFile.xlsx'); % <-- Insert your filename here

% Assign columns

PFD = num(:,1); % Photosynthetic photon flux density

Cc = num(:,2); % Chloroplastic CO₂ partial pressure

A = num(:,3); % Net photosynthesis rate

% Range of Cc values for curve generation

Cc_range = InsertRangeHere;

% Insert your estimated or fixed parameter values below

R = InsertValueHere;

O2 = InsertValueHere;

P = InsertValueHere;

e = InsertValueHere;

alpha = InsertValueHere;

theta = InsertValueHere;

Ka = InsertValueHere;

Kc25 = InsertValueHere;

Ko25 = InsertValueHere;

Ea_Kc = InsertValueHere;

Ea_Ko = InsertValueHere;

Vcmax = InsertValueHere;

Jmax = InsertValueHere;

T = InsertValueHere;

gamma_star = InsertValueHere;

DL = InsertValueHere;

% Calculate temperature-dependent kinetic constants

Kc = Kc25 * exp(-Ea_Kc / R * (1 / (T + 273.15) - 1 / 298.15));

Ko = Ko25 * exp(-Ea_Ko / R * (1 / (T + 273.15) - 1 / 298.15));

Km = Kc * (1 + O2 / Ko);

% Unique PFD levels

unique_PFD = unique(PFD);

colours = lines(length(unique_PFD));

% Prepare figure

figure; hold on;

% Storage for regression parameters

slope = zeros(length(unique_PFD), 1);

intercept = zeros(length(unique_PFD), 1);

% Plot experimental points and regression lines

for i = 1:length(unique_PFD)

idx = PFD == unique_PFD(i);

plot(Cc(idx), A(idx), 'o', 'LineWidth', 2, 'MarkerSize', 8, ...

'Color', colours(i,:), 'MarkerFaceColor', colours(i,:));

% Linear regression

p = polyfit(Cc(idx), A(idx), 1);

slope(i) = p(1);

intercept(i) = p(2);

x_fit = linspace(min(Cc)-1, max(Cc)+1, 200);

y_fit = polyval(p, x_fit);

plot(x_fit, y_fit, '-', 'Color', colours(i,:), 'LineWidth', 1.5);

end

% Plot FvCB curves

for i = 1:length(unique_PFD)

A_pred = zeros(size(Cc_range));

for j = 1:length(Cc_range)

A_pred(j) = farquhar_model(Cc_range(j), unique_PFD(i), Vcmax, Jmax, DL, ...

Kc, Ko, O2, gamma_star, alpha, theta, e, Ka);

end

plot(Cc_range, A_pred, '--', 'Color', colours(i,:), 'LineWidth', 1.2);

end

xlabel('C_c (Pa)', 'FontSize', 12);

ylabel('A (\mumol CO_{2} m^-^2 s^{-1})', 'FontSize', 12);

title('Comparison of Linear Regression and FvCB Model');

hold off;

% Estimate intersections between lines

x_int = zeros(3,1);

y_int = zeros(3,1);

x_int(1) = (intercept(2) - intercept(1)) / (slope(1) - slope(2));

x_int(2) = (intercept(3) - intercept(1)) / (slope(1) - slope(3));

x_int(3) = (intercept(3) - intercept(2)) / (slope(2) - slope(3));

y_int(1) = slope(1) * x_int(1) + intercept(1);

y_int(2) = slope(1) * x_int(2) + intercept(1);

y_int(3) = slope(2) * x_int(3) + intercept(2);

% Display estimated Gamma* and DL

Gamma_star_est = mean(x_int);

DL_est = mean(y_int);

fprintf('Estimated Gamma* from regression: %.4f\n', Gamma_star_est);

fprintf('Estimated DL from regression: %.4f\n', DL_est);

% Supporting Function

function A = farquhar_model(Cc, PFD, Vcmax, Jmax, DL, Kc, Ko, O2, gamma_star, alpha, theta, e, Ka)

Km = Kc * (1 + O2 / Ko);

Vcmax_prime = (Vcmax * Cc) / (Cc + Ka);

Wc = (Vcmax_prime * Cc) / (Cc + Km);

J = (alpha * PFD + Jmax - sqrt((alpha * PFD + Jmax)^2 - 4 * theta * alpha * PFD * Jmax)) / (2 * theta);

Wj = (J * Cc) / (4 * (Cc + 2 * gamma_star));

Vc = (Wc + Wj + e - sqrt((Wc + Wj + e)^2 - 4 * Wc * Wj)) / 2;

A = Vc * (1 - gamma_star / Cc) + DL;

end

**Supplementary Notes S3.** Stepwise Procedure for Parameter Fitting and Estimation of Γ* and D_L_ Using the Laisk Method through the FvCB Model.

1. Load experimental data: C_c_ (chloroplastic CO_2_), PFD (light intensity), and A_net_ (net photosynthesis). *Note that the method can also be applied using intercellular CO_2_ (C_i_) instead of chloroplastic CO_2_ (C_c_), assuming infinite mesophyll conductance* (gm **→ ∞**)*. In this case, the resulting estimates of Γ* and D_L_ would be apparent.*
2. Set the temperature and initialise constants: R, O_2_, e, K_c_, K_o_, E_a__K_c_, E_a__K_o_, K_a_.
3. Define initial guesses for six parameters: Γ*, D_L_, V_cmax_, J_max_, α, and θ (considering their temperature dependencies).
4. Define the cost function: mean squared error between observed A and modelled A.
5. Use a minimisation algorithm to find the parameter values that minimise the cost function.
6. Extract the optimised values of Γ* and D_L_, which represent the model-based implementation of the Laisk method using the FvCB framework.

**Supplementary Figure S1.** Response of mesophyll conductance (gₘ) to environmental conditions in sunflower (*Helianthus annuus* “Russian giant”).

Plants were grown in a controlled environment growth room at the University of Sydney, Centre for Carbon Water and Food (Camden, NSW, Australia). The growth room was set to a 16 h photoperiod, 25/17 °C day/night temperature, 75% RH, 49.7 Pa CO_2_ and 300 μmol photons m^−2^ s^−1^ provided by metal halide lamps. The youngest fully expanded leaves on four replicate plants (or five replicates for the temperature response) were used for gas exchange measurements, 5-7 weeks after germination. Mesophyll conductance was measured using a coupled leaf gas exchange and carbon isotope discrimination technique as described in Shrestha *et al*. (2019). Unless otherwise indicated, the leaf gas exchange chamber was controlled at 1300 μmol photons m^-2^ s^-1^, 40.0 Pa CO_2_ and leaf temperature at 25°C.

**FIGURE S1** | Mesophyll conductance (gₘ) in sunflower (*Helianthus annuus* “Russian giant”) as a function of three environmental factors. Panel (a) shows gₘ measured at four light intensities (300, 500, 700, and 1300 µmol photons m⁻² s⁻¹). Panel (b) shows gₘ at five temperatures (15, 20, 25, 30, and 35 °C). Panel (c) shows gₘ at four intercellular CO_2_ partial pressure. Experimental data points are shown individually, with dashed lines representing the fitted regression curves. The corresponding equations and R² values are displayed in each panel.

**Supplementary Figure S2.** Sensitivity analysis of the estimated parameters Γ* and D_L_ to mesophyll conductance (gₘ) in sunflower (*Helianthus annuus* “Russian giant”).

The influence of gₘ on the estimation of Γ* and D_L_ is shown through a sensitivity analysis conducted by varying gₘ between 0.5 and 10 µmol m⁻² s⁻¹ Pa⁻¹. The analysis was performed using the Excel-based tool developed in this study. Results illustrate the robustness of the parameter estimates to changes in gₘ within a physiologically plausible range.

**FIGURE S2** | Sensitivity of parameter estimation to mesophyll conductance (gₘ) in sunflower (*Helianthus annuus* “Russian giant”). Estimated values of Γ* (a) and D_L_ (b) are shown as a function of gₘ. The simulations were conducted at 25°C using the Farquhar model implemented in the Excel-based fitting tool developed for this study, with all other parameters held constant.

**Supplementary Figure S3.** Replot of the Yin et al. 2011, wheat A–C_i_ dataset at 25 °C: D_L_ and Γ* from the conventional Laisk method and our proposed approach.

We used the A–C_i_ dataset reported by Yin et al. (2011) for wheat leaves at 25 °C. They introduced a chlorophyll *a* fluorescence-based method to improve estimates of light CO_2_ release D_L_, termed R_d_ in their paper. The same dataset was later reanalysed by Yin and Amthor (2024) using an alternative method to the linear Laisk approach based on a non-rectangular hyperbola. In the present work we do not reproduce their analysis. Instead, we apply the conventional Laisk method with linear regressions and our proposed approach to the same dataset under both narrow and wide CO_2_ ranges, and we present replotted results to allow qualitative comparison with the method reported by Yin and Amthor (2024).

**FIGURE S3 |** Linear and non-linear Laisk analyses from wheat leaves at 25 °C. Redrawn using the wheat leaf dataset at 25 °C originally reported by Yin et al. (2011), following the re-plotting and interpretative framework of Yin & Amthor (2024). Net CO_2_ assimilation (A, µmol m^-2^ s^-1^) is plotted against intercellular CO_2_ mole fraction (C_i_, µmol mol^-1^). Three light intensities are shown, 100, 200, and 300 µmol photons m^−2^ s^−1^, encoded as yellow circles, orange squares, and dark-red triangles, respectively. The dotted horizontal line marks A = 0. D_L_ is reported as a positive magnitude (D_L_=R_d_). The symbol C_i_* denotes the apparent ɼ* inferred using C_i_. (a) Linear regression using a wide CO_2_ range. Straight line fits of A–C_i_ for each PPFD. Pairwise intersections of the regressions (black asterisks) are averaged to obtain estimates of C_i_* and D_L_. (b) FvCB model using the wide CO_2_ range. The same data fitted with the full FvCB model. The red asterisk marks the model-based intersection (C_i_*, D_L_). (c) Comparison between linear and FvCB using a narrower CO_2_ range. Black asterisks show linear-method intersections, and the red asterisk shows the FvCB intersection.
